# “You Never Exhale Fully Because You're Not Sure What's NEXT”: Parents' Experiences of Stress Caring for Children With Chronic Conditions

**DOI:** 10.3389/fped.2022.902655

**Published:** 2022-06-27

**Authors:** Stephanie Smith, Mary Tallon, Carrie Clark, Lauren Jones, Evalotte Mörelius

**Affiliations:** ^1^School of Nursing and Midwifery, Edith Cowan University, Joondaulup, WA, Australia; ^2^Perth Children's Hospital, Nursing Research Department, Nedlands, WA, Australia; ^3^School of Nursing, Curtin University, Bentley, WA, Australia; ^4^Kalparrin, Perth Children's Hospital, Nedlands, WA, Australia; ^5^Parent Representative, Banbury, WA, Australia

**Keywords:** parents' perspectives, stress, children, stress coping, chronic conditions, lived experience, qualitative

## Abstract

Children with chronic conditions are experiencing improved survival worldwide, and it is well-known that their parents are stressed. Yet, despite this knowledge, parents continue to experience stress. Our study explored the lived experience of parental stress when caring for children with various chronic conditions to identify opportunities to potentially reduce stress for these parents. This was an exploratory qualitative study using semi-structured interviews. To ensure appropriate research priorities were addressed, the study was co-designed with consumer and stakeholder involvement. Twenty parents were interviewed. Parents were recruited through a recognized family support organization for children with various care needs in Western Australia. Interviews were audio-recorded, transcribed verbatim, anonymized, and analyzed using Interpretative Phenomenological Analysis. Two superordinate themes were identified: (1) Gut instinct to tipping point included parents as unheard experts and their experiences of stress and becoming overwhelmed. (2) Losses and gains covered the parents' identity and relationship challenges and coping strategies with their children's unpredictable conditions. Parents' experiences of stress caring for children with chronic conditions can be applied to the Job-Demand Control-Support Model for occupational stress. Not only does this application provide a useful framework for practitioners but it adds a unique perspective that reflects the dual role of parents in caring for their children with chronic conditions as a parent but also a professional with a 24/7 workload. The parents' experiences highlight a need for improved support access, effective communication between parents and health care professionals, discharge preparation and information provision, and regular screening of parental stress with a referral pathway.

## Introduction

Chronic conditions, also known as long-term conditions, among children are rising (1) and refer to a wide range of conditions, illnesses, and diseases that tend to be long-lasting with persistent effects (2). In 2017–18, around 43% of Australian children aged 0–14 had at least one chronic condition, while 20% had two or more chronic conditions (2).

With progressions in surgery, treatments, and critical care, children who may not have previously survived have improved prognoses (3). Reviewing evidence of children with different diagnoses worldwide highlights the improved survival rates. For example, children with cystic fibrosis are expected to survive into at least their fifth decade in the UK (4). Similarly, registry data in Sweden found 1-year survival improved between 2004–2007 and 2014–2016 among extremely preterm babies (5). Children in the US with a severe form of congenital heart disease who previously succumbed to the disease within the first year of life are now surviving to adulthood (6). Likewise, children born with various major congenital anomalies are now predicted to survive up to 20 years (7).

The complex care that is required for the child's condition(s) is often provided by parents (8) at home (3) and can be emotionally demanding (9). Parents are responsible for advanced medical procedures (10), as well as daily caregiving exceeding that of healthy and typically developing children. This increases the risk for poor health, psychological distress and exhaustion, lower quality of life, and sleep deprivation (11–14). Altered saliva cortisol levels have also been noted with parents of sick children in hospital, reflecting the chronic stress state these parents are under (15). Parental stress has been defined as the level of psychological distress one experiences while trying to meet the demands of the parenting role (16). It has also been described as the discrepancy between the resources required for the parental role and the perception of being able to cope with them (17). Therefore, parental stress requires more attention to find interventions to prevent the risk of stress-related complications that subsequently can affect the care of the child (15) and negatively impact on the child's behavior and cognitive development (18, 19).

It is recognized that different chronic conditions present common challenges to families (20). Yet, previous research into parents' experiences has also tended to focus on particular child chronic conditions (20, 21), which limits an understanding of what is known across different diagnoses (20). Leeman et al. (20) argued that this limits the potential to generalize knowledge of the effects of family functioning on the well-being of chronically ill children. Similarly, considering that some children often have more than one chronic condition (2) it is therefore imperative to find commonalities and support possibilities that suit parents regardless of the child's disease or diagnosis (20). Despite fathers often being involved in caregiving, they are often underrepresented in research (22). Fathers are important for the family system and how the family system handles stress. Therefore, there is a need for their views on parental stress. This study sought to provide a detailed understanding of the issues faced by mothers and fathers caring for children with various chronic conditions in Western Australia and how they ascribed meaning to their experiences.

## Methods

### Design

This study used a qualitative method with an Interpretative Phenomenological Analysis (23). Disconnection between researchers' study aims and research end-user needs is a major cause of research waste (24). Meaningful involvement of end-users can address this research waste (24). Co-design has been described as meaningful end-user engagement in research design and includes instances of engagement that occur across all stages of the research process and range in intensity from relatively passive to highly active and involved (24). This study was co-designed with parents of children with chronic conditions to explore their needs and research priorities. The research idea was presented to a consumer advisory panel that were parents caring for children with chronic conditions and a youth advisory panel of young people with various chronic conditions. The research idea, design and recruitment process were presented and received positively by both groups. Parents acknowledged more support is needed for parents caring for children with chronic conditions, and the youths with various conditions provided feedback that their condition can have an impact on the family as a whole. In particular, LJ, (an experienced parent representative) was a member of the research team and was involved throughout the research process. LJ and consumers from the advisory panel advised on the recruitment strategy and the benefits of collaborating with a recognized family support organization that families knew the person contacting them about the study and support was available for parents after the interviews, if required. LJ and consumers from the advisory panel were involved in developing the interview guide to ensure relevant questions were asked. LJ was involved in creating the study documentation including the study invites and parent information and consent form. In all cases, LJ ensured that interview questions and the study documentation were worded appropriately for parents caring for children with chronic conditions. LJ consulted on the findings and was involved in the study recommendations. LJ had also been a co-investigator in the study approval applications and grant application that funded the research. The co-design process was achieved through team meetings and over email throughout the course of the research. This collaboration has been described previously (25). The study documentation was also reviewed by the third author (CC) and her team at the family support organization, who have a wealth of experience working with many families providing advice, guidance and support raising children with various needs.

### Participants

In this study, a “parent” was a person with the care responsibilities of the child. A purposive stratified sample was recruited to ensure a balanced representation of parents caring for children with chronic conditions covering three child age groups defined in the literature as (1) birth to preschool 5 years (26), school-age 6–12 years (27) and adolescence 13–19. Deatrick and Faux (27) mention 13–18 for adolescents; however, the World Health Organization (WHO) definition includes adolescents to the age of 19 was also taken into account (28).

Inclusion criteria were parents of a child, with at least one chronic condition, aged from 0 to 19 years, diagnosed/started treatment within the last 5 years, and was 6 months post-diagnosis/treatment. The criteria were reviewed with the consumer advisory group, LJ and CC and these timeframes were considered acceptable. CC organized for eligible participants registered with the family support organization to be contacted. Interested participants were emailed the parent information and the consent form by the family support organization and contacted the research team for further information or requested to be contacted by the research team.

We aimed to recruit up to 24 participants (eight parents in each child age group). In line with Smith et al. (29) recommendation of between six and ten participants, it was decided to go for the middle ground on this recommendation and aim for up to eight participants in each age group. This decision also covers the freedom on the decision of sample size as proposed by Smith et al. (23). The sampling approach and a larger number of participants have been used with other IPA studies [e.g., (30)]. Thirty-three parents were invited to participate, one declined, 12 lost contact, and 20 (10 mothers, 10 fathers) took part in the study. A total of six participants were three married couples. Four of the participants lived in regional Western Australia. Table 1 provides the demographic overview and participant profiles. Pseudonyms for the parents have been applied and are outlined in Table 1.

**Table 1:** Group demographics.

	**Group 1**	**Group 2**	**Group 3**
	**Birth to preschool**	**School-age**	**Adolescent**
	**(0–5 years)**	**(6–12 years)**	**(13–19 years)**
Relationship to the child and pseudonyms	4 Mothers •Alice •Eleanor •Kara •Tess 4 Fathers •Aiden •Josh •Miles •Steven	2 Mothers •Amber •Polly 3 Fathers •Markus •Ryan •Todd	4 Mothers •Emily •Libbie •Melody •Sally 3 Fathers •Daniel •Greg •Theo
Age (ranges)	31–44	37–63	43–55
Number of parents with more than one child with chronic conditions	1 Mother	1 Mother 3 Fathers	1 Mother 2 Fathers
Ethnicity	8 Caucasian Australian	4 Caucasian Australian 1 Caucasian British	1 Aboriginal and/or Torres Strait Islander peoples 1 Asian Indian 4 Caucasian Australian 1 Caucasian British
Employment status	4 full-time work 2 part-time work 2 full-time carers	2 full-time work 3 full-time carers	1 full-time work 2 part-time work 4 full-time carers

Participating parents named a range of diagnoses when asked to describe their children's health conditions. Most included more than one health diagnosis and required specialist care from two or more specialist health teams. Additionally, many children had neurodevelopmental disorders. The children's health conditions and neurodevelopmental disorders are categorized for anonymity purposes and are presented in Table 2 in accordance with the Australian Bureau of Statistics categories for chronic conditions (31).

**Table 2:** Participants' children's health conditions according to age groups.

**Age group**	**Classification of health condition**	**Named health condition**
Group 1: Birth to preschool (0–5 years)	Congenital/syndrome.	Charge syndrome. Craniofacial. Mandibulofacial dysostosis. Microphthalmia.
	Developmental.	Cerebral Palsy. Gross developmental delay. Microcephaly.
	Gastroenterology & Nutrition.	Gastrointestinal motility disorders. Percutaneous endoscopic gastrostomy feeding.
	Heart disease.	Absent subclavian/lymphectasia. Congenital heart disease.
	Neurological function.	Epilepsy. Hydrocephalus.
	Respiratory/airway.	Chronic lung disease of prematurity. Laryngomalacia. Tracheomalacia. Tracheostomy.
Group 2: School-age (6–12 years)	Congenital/syndrome.	Fetal Alcohol Syndrome Disorder. Cleft palate.
	Developmental.	Cerebral Palsy. Gross developmental delay. Microcephaly.
	Mental health.	Anxiety.
	Neurodevelopmental.	Autism Spectrum Disorder. Attention deficit/hyperactivity disorder.
	Neurological function.	Epilepsy.
Group 3: Adolescents (13–19 years)	Chronic pain.	Complex regional pain.
	Congenital/syndrome.	Rare multisystem syndrome. Scoliosis.
	Developmental.	Global developmental delay.
	Gastroenterology and Nutrition.	Gastrointestinal motility disorders. Percutaneous endoscopic gastrostomy feeding.
	Mental health.	Anxiety.
	Neurodevelopmental.	Attention deficit/hyperactivity disorder.
	Neurological function.	Refractory seizures.
	Respiratory/airway.	Cystic fibrosis.

### Data Collection

Data were collected using a semi-structured interview guide. Sixteen interviews were telephone interviews, and four were face-to-face, with three conducted in private consultation rooms at the hospital and one in a private neutral place. Informed consent was obtained before the interview, and participants completed a demographics form with the researcher before the interview started to provide context to the interview and overall sample.

Interviews were semi-structured, audio-recorded, and conducted by two experienced female qualitative researchers, SS and MT. Any sensitive or emotional issues that arose were handled sympathetically and confidentially. If a parent became emotional, they were given time and were advised they could skip questions if needed. A few of the parents became emotional but wanted to answer all questions.

The interview questions guided the interviews and were open to exploring issues that participants raised themselves. Probes were also included in the interview guide to assist for further depth. The questions were framed around the following topics: stress, coping, relationships, identity, recommendations/improvements to parental stress. The interviews ranged from 26 min to 3 h.

Interviews were transcribed verbatim by the interviewer, and identifying information was removed. The interview guide is available as Supplementary Material.

### Data Analysis

Interpretative Phenomenological Analysis (IPA) (23) guidelines were followed. IPA is an inductive approach and was selected as it emphasizes sense-making, especially with experiences of major significance (23, 32). Three researchers, SS, MT, and EM, were involved in the analysis process. SS led the analysis and read and listened to each interview many times to become familiar with the data. Each transcript was analyzed in its entirety before moving on to the next due to the idiographic nature of IPA. Initial notes and thematic labels were recorded on the transcript and then summarized in a notebook which also acted as a reflexive journal. Each transcript was also read by MT and EM. Regular team meetings took place to review each transcript in turn. During the team meetings, each researcher's initial thoughts, comments, and comparisons on the transcript and significant or interesting aspects, both descriptive and interpretative, were discussed. As more transcripts were reviewed, comparisons were also discussed and noted separately but drawn on in the later stages of the analysis. The research notes/themes discussed at team meetings were summarized, which aided the analysis and dealing with the large data set. These resulted in themes illustrating key aspects of the experience for that participant.

NVivo (33) was used to manage the data. Comparisons of convergence and divergence between transcripts were made in NVivo, and then visual representations were used to identify recurrent themes, connections, and interrelationships that reflected shared and different experiences of parents caring for a child with chronic conditions. This was an iterative process returning to the transcript, and reflections/notes made, and themes were adapted, discarded, or reinstated. The analysis process was reviewed regularly with SS and EM and continued in the writing of the findings as commonalities and differences between participants became clearer. Interpretations were reviewed by all authors.

## Results

Two superordinate themes were derived. The first superordinate theme, “gut instinct to tipping point,” describes the parents' experiences of not being heard on their child's condition and becoming overwhelmed and is comprised of two subthemes “the unheard experts” and “stressed and overwhelmed.” The second theme, “losses and gains,” describes the parents' identity, relationship challenges, and coping strategies through the subthemes “identity and relationship effects” and “coping with the unpredictable.”

### Gut Instinct to Tipping Point

This theme describes the parents' experiences of feeling unheard by healthcare professionals despite feeling that something was wrong with their child to reaching a point where the lack of support and the responsibility on parents became stressful and overwhelming.

#### The Unheard Experts

For most parents, obtaining their child's diagnosis proved difficult. Many described having a “gut instinct,” “intuition” or “inkling” that something was wrong. Sally describes this innate detection despite having no prior experience: “…[child] wasn't meeting her milestones…just that gut feeling something wasn't right. Even though [child]'s our only child.”

Attempts to express their concerns to healthcare professionals resulted in many feeling dismissed. This was reflected in Emily and Greg's descriptions of themselves, ‘I'm just a parent' (Greg), ‘I'm just a Mum' (Emily), to later find out their instincts were right:

I thought he had [infection]…because I could hear it in his cough, I was ignored…and two months later he was found to have [name of severe infection] (Emily)

…they said, “ah, this is a seizure.” I said, “no it looks the same [as the previous life-threatening incident]”…they didn't want to listen to me. So, an hour later they said, “oh this isn't a seizure.” (Greg)

Both Emily and Greg illustrate how most parents drew on their senses and were able to read their children through their sounds and/or visual cues.

Feeling unheard by healthcare professionals resulted in some parents questioning themselves. Eleanor's action of pursuing and listening to her instincts about her daughters feeding issues despite seeing several different GPs proved lifesaving:

I thought I was going crazy…everyone was saying…it was all [in] my head…I got to a point where we rushed her to the hospital…we got told…her [condition causing the feeding issues]…she actually had this from birth…they reckon within a matter of days she would have been dead.

Not feeling heard resulted in some parents resorting to collecting clinical evidence by keeping samples, videos, or photos to show healthcare professionals or only feeling heard once their child's condition was witnessed by a healthcare professional. Eleanor described her emotional release of her child finally being diagnosed as ‘howling like an animal'. Her primal reaction highlights her relief that her child had a diagnosis but also a release that she had finally been acknowledged and heard.

Feeling validated was noted with parents with children with more specialized conditions in comparison to parents whose children had multiple complex needs. Therefore, impacting parents' stress levels. For example, Josh described his unique relationship with the consultant that made him feel supported and heard: “…our head consultant…is exceptional…If we have a concern, we've got his…number to ring and you know, he always answers.”

#### Stressed and Overwhelmed

Stress for parents was referred to in various ways from having “gray hair,” “free-falling” implying having a lack of control, the “constant worry” and being on “high alert.” Stress was also described as: “it takes your brain.” Melody highlights further her cognitive issues and now feels she represents a different person: “I have gone from remembering everything and functioning like a normal person to having to write everything down.” Similarly, Aiden highlights how essential items for his child were easily missed since his child's condition was constantly on his mind: “…we walk out of the house, and we've got everything we need…but we forgot nappies the most common simple thing…but you are so focused on…just her breathing.”

The continual setbacks with their child's development and living with the constant unpredictability of the child's condition were frequently described as a “rollercoaster ride.” Kara highlights this ride of ups and downs: “he [child] achieves something, and he gets so excited and then he goes so far backwards…in the last few days really picked up his confidence with walking…we are on a massive high at the moment” (Kara).

Shallow breathing is known as a typical stress response, and Theo implies this regarding dealing with the uncertainty with his child's condition and their various procedures and hospital admissions: “part of the stress…of [child] just being sick or having operations continually…you never exhale fully because you're not sure what's next, what's coming.” Due to the complexity of the children's chronic conditions, many had multiple medical conditions with various diagnoses occurring over many years and/or unknown conditions where healthcare professionals were even baffled. At times of delayed diagnoses, parents described feeling like their child was being experimented on: “they started to use her as a guinea pig because they didn't know what was wrong” (Greg). Similarly, Kara described: “a lot of it was just a bit of a trial and error which was always a bit scary.”

Physical stress included moving and lifting their child due to not receiving appropriate equipment, which became difficult as the child got older or having painful knees and back pain. Financial stress was also mentioned due to parents having to give up work or the difficulties with receiving financial support: “[Government agency to assist with payments] is a pain…The [disability support organization] is even worse…It's not a good…fun life I wouldn't wish on anyone” (Greg).

The situation became overwhelming because of the constant struggle and unpredictability along with a lack of time-out and support. The parents did not receive much respite, and for those that worked, annual leave was often used for the child's numerous appointments or procedures or when the child was sick, with work described as a place to relax. Some parents had to leave their jobs or wanted to work, but it was not possible due to the care required for their children. A few reported that their partners had reached burnout with some suffering mental health issues, and they not only had to support their child but also their partner and other children. Often one parent had to work to survive financially, leaving the other with full caring responsibilities. Simple everyday events like going for a coffee were difficult due to the amount of equipment required for some of the children. Parents described feeling restricted as they could not travel far in case they were needed and did not have time for themselves even when the child was at school: “I do get a physical break from her but just not a mental break” (Melody).

Many parents felt unsupported by healthcare professionals and uninformed about what was happening with their children. Not being included in multidisciplinary team meetings, turning up for appointments to be turned away, or seeing a fellow/registrar and told to come back to see the consultant when they had been advised they were seeing the consultant or being given an early morning appointment for their child when they had to travel a distance to attend and pack the amount of equipment needed to accompany their child, was frustrating. Obtaining any support was described as a constant battle. The word “fight” was often used, and Greg's repetitive use emphasizes how constant this was from various aspects: “fight to get the medical stuff, you got to fight to get the support required and the fight to get a financial…enough to survive.”

A lack of communication between the various healthcare professional teams/departments was often mentioned, which meant the responsibility of keeping everyone up to date fell on the parents. Many developed an unhealthy control on their child's care due to this responsibility which could impact their own health and the feeling of being overwhelmed. Daniel termed himself and his wife as “helicopter parents” when their child was admitted to hospital, suggesting they did not feel comfortable with others looking after their child and needed to keep close attention to monitor their child's state, highlighting a lack of trust. This close attention continued at home for many, and Amber's emotional reaction reveals the constant vigilance for her and her husband: “We never leave him with anyone else…if we go out, he always comes out with us…you're sort of on high alert all the time, and I think it is quite stressful [crying].”

The parents were their child's advocate, but when it came to who was advocating for the parents, many were often left to support themselves, with most not being offered psychological support:

I don't think they really offer us any support as such. And it's not very often that they check in…to see how the parents are going…They could probably improve on just like, general well-being of the parents of the kids to make sure they've got enough mental stability or security…to help with the kids (Steven).

Often reported was needing an advocate to support parents with the finance support applications and to know what support services are available to them. Emily described the benefits of having an advocate when attending a multidisciplinary team meeting:

when [nurse] came to this meeting I already told [nurse] everything I was thinking but I was so put on the spot I couldn't find my words…[nurse] said almost verbatim what I said to [nurse] half an hour earlier. And I just, I've never felt so supported in…16 years.

Some of the parents had mixed emotions when learning that their child had been eligible for support. They were happy that their child was now in receipt of support but also angry that their child had missed out on such supports that may have aided their development. Libbie recommended needing a handbook that detailed the support and resources available for parents.

Feeling overwhelmed was often linked with a lack of sleep from worry or interrupted sleep to care for their child. The majority did not sleep well, describing their sleep as terrible. Sleep is a time to rest and recharge, yet the responsibility for most did not stop: “I'm trying to sleep but then I'm also trying to stay half-awake listening out for [child]” (Miles).

Most had no time to process their situation and felt stuck in a situation where they had no choice. The multiple stressors parents often experienced together and the repetitiveness of the stressors, it is unsurprising why most became overwhelmed. Often it was the little things that tipped parents over:

…we wanted to order these little stubby holders to sell you know for [child]…[partner] said…‘I'm sorry we are not going to be able to get them done on time' and I just burst into tears. And I am not one usually to get too emotional…I realize I am just overwhelmed with everything (Alice)

### Losses and Gains

This theme describes the parents' identity challenges and the impact of having a child with chronic conditions on their relationships. Their coping strategies, both negative and positive, are highlighted.

#### Identity and Relationship Effects

Many of the parents experienced having an unexpected role that they had not imagined:

…a 13-year-old girl would not normally have to be changed by their father…she's going through obviously periods…it's not a thing that most fathers would have to deal with…we've been doing it since…she's been a baby…it's sort of natural. But when you think about it…it's very unnatural (Daniel)

You become a nurse, and you become a doctor, and you become a pharmacist (Libbie)

There's a sense of sadness in Daniel's response to his and his daughter's situation. Similarly, many described living a “warped normality.” Libbie's response implies that her role as a mother was sidelined. Likewise, some of the mothers described only fulfilling the mother role when their second child was born (without chronic conditions).

Family life was often split between hospital and home, with families torn apart, for example, one parent in the hospital with the child, and the other with the siblings: “…juggle living separately with two older girls, trying to provide a normal sort of stable living conditions for them without mum and the newer sister being home…we've never lived really as a family unit” (Josh).

Many described having altered relationships with their other children due to the child's condition. Due to a lack of support many encountered, some parents relied on the siblings to help with the child's care. Siblings were described as a “mother-figure” or even registered as a “young carer.” Many felt worried and guilty about the lack of time they had with their other children: “he [sibling] missed out on a lot of things because [child] has not been well” (Miles).

Parents experienced their identities being threatened regularly. Libbie described this as “living life in a goldfish bowl” where their lives are on show for all to judge:

I got told [by peers] “oh you just stay at home [not working]; you don't do anything.” And the perception is that like they don't comprehend like anything can happen with [child] and also the amount of appointments…They…have no idea at all (Polly)

Many felt exposed to the various healthcare professionals and carers that were involved in their child's care. Some parents refused night-time carers as they needed to have their own space and time as a family. Similarly, some mentioned feeling judged if they left their child during their hospital admission. Emily was the only parent to describe receiving respite during her child's hospital admission, and building up to this had taken her years in preparing her child and something she felt ashamed of sharing:

[Nurse] “Oh you're here today?” [in a judging tone]. That really does have an element of “Oh you weren't here yesterday”…I would never say this out loud to anybody but the only real respite I get is when [child] is in hospital more so since he's been, had the ability to do a bit more for himself.

A few of the parents embraced new aspects to their identity, and Aiden described himself as a “semi-expert” with his child's condition, highlighting that some parents felt empowered. Similarly, some felt it had brought them closer to their partners and family. However, many struggled to retain a sense of who they were before their child's condition. Those that were still able to work or study implied having dual identities and a need to relate to a part of themselves that was separate from their child: “So, when [child] gets dropped off at day-care, I become Tess again.”

Others lost and grieved their previous self and who they used to be. Many stopped working, traveling, or doing sports or hobbies once enjoyed as their child's care and family took precedence: “I don't even know what favorite foods I have…I've really lost sight of what it is that I used to, before all this…I don't have a sense of self anymore” (Markus).

Finding themselves again was reported as an impossible task due to the demands required in caring for their child. Some described having an “intertwined identity” implying their identities were now merged with their child: “whatever mood she's in…will reflect in my mood, so when she's cruising, I cruise…Her lungs are my lungs…it's almost…host-parasite kind of” (Libbie). The host-parasite metaphor resembles the child relying on and living off the parent, which in Libbie's case, she was her child's main carer. Parasites can represent harming the host and changing its behavior and many parents described negative and positive changes. Negative changes ranged from becoming “old, grumpy and stressed out,” having a low tolerance, and not prioritizing themselves, which can end up being detrimental to parents. Positive changes included becoming more patient, accepting, and being involved in the disability community.

Most experienced losing touch with friends and described that family members were often too worried to look after their child or had difficulty understanding their child's condition, promoting feelings of isolation. Todd described his fractured relationship with his mother ignoring his child's condition: “[Child] had such a huge allergy…they [Grandparents] would invite us over, and they would say there will be no dairy…she'd [Grandmother] bring out the meringue…with cream.”

For some, there was a need to identify with others in similar situations who would understand them. The common experiences with their children made some feel as if they were in the same boat and were understood which was lacking from their other relationships. Yet, many struggled to attend groups as Greg described: “a lot of stuff they do [support organization]…is all female orientated. And if they do a Dad event, it's…too far away.” Others could not find a group specific to their child's complex conditions as Sally described: “we tried a couple of different groups, and we didn't seem to fit…And people could exclude you quite quickly.”

Sexual relationships were impacted as many parents slept separately from their partner, with one parent often sleeping with the child and/or they were too tired and stressed from the child's constant care. Some reflected that their living situation was more like “housemates,” implying their identity and closeness as a couple was lost. Regularly mentioned as being like “two ships in the night” or “tag-teaming,” reflecting the lack of time couples had together. Only one participant had separated, but the majority described their marriages as strained.

#### Coping With the Unpredictable

Over time, parents described adapting to what was considered their new normal and for some became comfortable or controlled what they could with the unpredictability of the children's conditions:

you have a set amount of seizures and a certain behavior during the day, and you call that normal (Daniel)

I go to sleep in…a tracksuit…if I've got to get up and rush into emergency, I don't have to take those minutes to…put on decent clothes…Not that he's going to…go to hospital every night…but he can just get sick like that [clicks fingers] (Emily)

Various coping strategies were employed by the parents to deal with the challenges they faced. Humor was regularly used in describing their experiences. Parents were under immense stress dealing with traumatic experiences concerning their child's conditions. Laughing and humorously describing their experiences not only presented a lighter perspective but was also a coping strategy and possibly a way of protecting them from the emotional pain of their reality: “I choose to laugh at a lot of situations…I find humor helps” (Libbie).

In coping with their ordeals, some of the parents went on a “mission” to get the appropriate help for their child and to also help other families and ensure that they did not have the same experiences as they had encountered:

I started then, not only fighting for our family, but I started fighting for other families as well to get support…we actually won the battle for [condition] to be on the [disability support organization] list. So now, families can now get the support and the children can now get the care that they really deserve. (Eleanor)

Strategies included celebrating the child's achievements, fundraising for support, getting out in nature/time with animals, undertaking regular exercise, meditation, massages, talking with psychologists and counselors, or using social media to be connected to support groups and/or blogging their experiences, and listening to podcasts. Negative coping strategies included overeating, staying up late to have some me-time, a lack of exercise, or shouting and yelling mostly at their partner to deal with their stress. Many were worried about the future, especially regarding what will happen to their child when they are no longer able to care for them. Some of the parents started to prepare their child where possible: “[Child] can do everything…I call it…in case I get hit by a bus plan. Because if…I'm in hospital unconscious I'm kind of the only one that can look after him” (Emily).

Many focused on the positives the situation provided from meeting families and healthcare professionals they would not have encountered and felt opened to another world. Finally, there was evidence of balancing the bad with the good in accepting their situation as exemplified by Ryan: “It has been…very tough, toughest thing I have ever done by a long, long shot, but also very enlightening.”

## Discussion

The research explored parental stress caring for children with chronic conditions. It was revealed that parents instinctively knew when something was wrong with their child yet struggled to be heard by healthcare professionals. The parents experienced immense stress in many areas (e.g., emotional, physical, and financial) from a lack of support, often resulting in feeling overwhelmed. Caring for a child with chronic conditions challenged the parents' identities, relationships, and coping strategies. To explain the complexities surrounding parents' caring for children with chronic conditions, the Job Demand-Control-Support model on occupational stress (34) may assist as its theoretical suggestions have been recommended to cover parents' experiences of caring for an ill child (35). Adapting the model to parents' experiences highlights this vulnerable group.

Job demands refer to the workload and have been operationalized mainly in terms of time pressure and role conflict (36). Parents provided full-time dependent care for their children which can be compared to work (35). In this study, the parents' job demands included a considerable workload, including attending multiple appointments and hospital admissions for their child, dealing with crises related to their child's condition, and managing their child's care. Their responsibilities impacted their identity and relationships with others. Many parents did not have respite, or get enough sleep, with self-care often non-existent. Many felt dismissed by healthcare professionals and uninformed about their child's condition and support services available to them which only added to their stress and unsatisfactory experiences. The various stressors that parents faced often occurring simultaneously, repetitively, and/or following on from each other without a break, resulted in many becoming overwhelmed. Research often shows gender differences in parents' stress experiences caring for a child with a chronic condition, with mothers reporting higher parental stress than fathers (37). This was not evident in the current study, but some of the fathers were dealing with extraordinary situations that most fathers of healthy and typically developing children would not encounter, especially in relation to caring for their daughters (e.g., changing their adolescent daughter and dealing with their daughters' periods) and how this situation differed from the norm.

The current study highlights that stress for parents ranges from stereotypical descriptions such as gray hair to a lack of control, constant worry, and being on high alert. Physical stress, cognition issues and poor sleep were also linked to parents' experiences of stress. Previous stress descriptions focus on the psychological aspects [e.g., (16, 17)]. The current study highlights that parents also experienced physical stress from carrying or lifting their child that got worse when the child got bigger. There is a need to review definitions of parenting stress in the literature and propose a concept analysis is required for future research [e.g., (38, 39)]. Having a clear definition ensures that we are all talking about the same thing. How we define parental stress will also impact how this area is researched and how interventions are planned. Regular screening of parents' stress and a referral pathway may prevent the development of longstanding stress.

Job control refers to the person's ability to control their work activities (36). In the current study, job control was limited for the parents. The child's unpredictable conditions made some parents feel a lack of control. However, with time many of the parents adapted and became comfortable with their new normal or developed strategies in preparing for events. Frequently mentioned was having an innate instinct when something was wrong with their child. This was described as a gut feeling or intuition and/or supported by reading their child's cues through their sounds or visual appearances that acted as signs that something was not right with their child. Yet, many struggled to be heard or listened to by healthcare professionals, which has also been noted in parents' experiences of children's fever [e.g. (40)]. In other research, instinct has been described as a sense of alarm (a feeling that something is wrong) or a sense of reassurance (a feeling that something is right) (41). There is a paucity of studies focusing on parents' instincts, yet those that exist have indicated that parents feel reassured when parental intuition is taken into consideration with healthcare professionals instead of criticized [e.g. (40)]. To gain control, parents need to be encouraged and empowered to trust their instincts by healthcare professionals, especially if their child has chronic healthcare needs. Failing to engage parents can disempower them and can jeopardize the child's care (42). As the current study highlights, parents and healthcare professionals need to work in collaboration so parents can gain and experience control. It is therefore critical that parents should be involved in shared decision-making about their child (43), and be seen as competent collaborators (44). However, parents can learn how to control their child's condition and deal with life-threatening situations and life-saving equipment, but they cannot control for unpredictable situations caused by healthcare professionals or other supporting organizations.

Job support refers to the social support within the workplace (45). Many of the parents lacked job support which in this context was with healthcare professionals, partners, friends, family, and support services. One father raised the issue that supports available were mostly female orientated. Similar findings have been noted in research focused on fathers looking after a child with life-limiting illnesses (46, 47) and chronic conditions (48). Some had supportive partners whilst others highlighted the pressures parents are under with partner burnout or mental health issues, leaving the responsibility to one parent. Many lost friends and family due to others not understanding their circumstances. It was clear in the findings that the child's condition affected parents' identity and relationships with others, especially with partners. Relational conflict has been noted as distressing for spousal carers in other health areas (3). In the current study, many described strained relationships with their partner and a loss of intimacy for some. Similarly, living with a child with a chronic condition has been shown to place parents at a risk of marital breakdowns (48), which can ultimately impact the care of the child if the main carer becomes overburdened with the loss of support. Other research has highlighted that parents need to accept new identities and let go of old identities, which may aid spousal relationships (49). Yet, for some parents in this study, holding on to their previous identity aided coping, with others grieving a lost previous self.

Previous friendships and family support were often mentioned in the current study in terms of feeling isolated. Parents may also experience increased stress from their usual informal support due to family and friends not understanding their situation (50), as also noted in the current study. The importance of peer support is widely recognized (51) but difficulties with friends and family are rarely mentioned (30). Future research is recommended to include support for friends and extended families and guidelines on how they can support the families.

Many of the parents in the current study recommended needing an advocate to help them through the systems and inform them of supports available for their child and for them. Similarly, it was noted that parents who had children with more specialized care received more satisfying support and were often empowered in comparison to parents with children with multiple complex conditions and require care from several specialists. This highlights a need to review more specialized conditions and their processes to aid the less specialized conditions. Evidence exists that greater access to respite care, peer and financial support can help to reduce the overload on families (52).

## Strengths and Limitations

The study was co-designed with consumer and stakeholder engagement. This ensured that important research priorities for families were addressed, and recommendations and further research enquiries remain targeted to parents' needs. Having a parent representative as part of the research team throughout the study provided essential, valuable, and meaningful expertise from the parent's viewpoint. Collaborating with the support organization may have aided the recruitment process as parents were contacted by someone they knew about the research, and the decision to contact or be contacted by the research team was left to the parent. Support for parents was also available from the family support organization after the interviews, if needed. Working with the support organization may have aided the equal numbers of fathers being recruited. Three researchers were involved in the analysis highlighting the rigor of the process. This study describes in detail the research co-design process that has been noted to be widely used but seldom described (24). It includes a shared collective from a parent representative, a family support organization, and advocates from parents in the consumer advisory panel in collaboration with researchers with nursing, pediatrics, and health psychology backgrounds.

The heterogeneity of the sample in terms of parents' age, gender, child's age group and condition may be considered to contrast IPA's homogeneity stance. Yet, during analysis, it was apparent there were no significant differences across these areas. We have therefore treated the sample as a whole during writing up as other IPA studies have (e.g. 30). We only interviewed parents who were English speaking, which may limit the applicability of the findings. Our inclusion criteria proved difficult when recruiting parents in the older child age groups as many of the children were diagnosed more than five years ago. To note, most of the parents were married apart from one parent who was separated.

## Clinical Implications

Despite this topic being a well-researched area, the fact that parents are still experiencing stress highlights that more targeted interventions are needed to support parents to gain control over their situation. Targeted interventions should include stressors that are often considered being minor and therefore overlooked. These stressors include but are not limited to canceled appointments, long travels to the hospital, lack of parking space, or frequent visits to different specialists. Such stressors may cause the tipping point since they are out of the parents' control. It was noted by both interviewers that many of the parents had stated that whilst the child's care is paramount, the parents are not focused on, and for some, this had been the first time anyone had enquired how they were doing. Clinical implications suitable for parents regardless of the child's disease or diagnosis concern the need to strengthen their control and the collaboration and shared decision-making between healthcare professionals and parents. The following areas of development may include:

### Support Access

Parents of children with chronic conditions need V.I.P. access to the healthcare, either physical or online, to decrease the time spent at each appointment/visit to the hospital. A specialist clinic that could be nurse, psychology, social work led, or a collaboration of the disciplines could address the advocate needs of parents and signpost them to psychological services/counseling and assist with obtaining home services including house cleaning, suitable equipment, and help with applications to support avenues. A focus on coordinating care for the whole family could better support parents to connect to a range of specialist, primary health care and local community services for wider support. In particular, targeted support for fathers is required. Male representatives are recommended to be available for the fathers in family support organizations. Father events need to be accessible for all or arranged in various locations so that all fathers have the opportunity to attend.

### Improved Communication Between Parents and Healthcare Professionals

Further practice in this area could be provided with supervised training with simulated/professional patients (often used in simulation laboratories) or mentorship. Similarly, further review is required on improving parents with children with multiple conditions in comparison to parents with specialized conditions to support and empower all parents. Application of strength-based models of care based on partnership, interdisciplinary communication, collaboration between families, and healthcare professionals that extends across healthcare settings would support this.

### Discharge Preparation and Intermediate Care Units

Preparation before going home can strengthen parent's control of handling the child's condition by themselves. An online handbook/guide covering support organizations, contact details and links to services would assist parents. In agreement with the parents, discharge preparation should also include special information to friends and relatives on how to best provide support to the particular family.

### Regular Screening of Parents' Stress and a Referral Pathway

Parents need to be regularly screened by healthcare professionals for their own well-being and for their children's care. This may prevent the development of longstanding stress and ensure earlier intervention to prevent parents reaching a tipping point.

## Conclusion

Children with chronic conditions are experiencing improved survival and are cared for at home by their parents. This study shows a gap between parents' needs and the care and support provided by the healthcare. The high demand parents are under with their various responsibilities, with low control and a lack of support may create stress responses resulting in psychological or physical health problems. On the other hand, strong support and more control may prevent parents' stress and require more targeted interventions.

## Data Availability Statement

The datasets presented in this article are not readily available because the data are based on sensitive information from parents' caring for children with chronic conditions. Requests to access the datasets should be directed to the corresponding author.

## Ethics Statement

The studies involving human participants were reviewed and approved by Child and Adolescent Health Service Human Research Ethics Committee (RGS0000003233), and reciprocal approval was obtained from two universities. Research Governance approval was obtained from Perth Children's Hospital. The participants provided their written informed consent to participate in this study.

## Author Contributions

SS, MT, CC, LJ, and EM made substantial contributions to conception and design of the study, involved in revising the manuscript critically for important intellectual content, gave final approval of the version to be published, and agreed to be accountable for all aspects of the work in ensuring that questions related to the accuracy or integrity of any part of the work are appropriately investigated and resolved. SS, MT, CC, and EM involved in the recruitment of participants. SS, MT, and EM made substantial contributions to the analysis and interpretation of data. SS and MT interviewed and transcribed interviews they conducted. SS involved in drafting the manuscript. All authors contributed to the article and approved the submitted version.

## Funding

The research was funded by the Perth Children's Hospital Foundation Seeding Grant #9889. The research was supported by the School of Nursing and Midwifery at Edith Cowan University and the Nursing Research Department at Perth Children's Hospital in Western Australia. The professorial position for EM was supported by Perth Children's Hospital Foundation.

## Conflict of Interest

CC was employed by Kalparrin. The remaining authors declare that the research was conducted in the absence of any commercial or financial relationships that could be construed as a potential conflict of interest.

## Publisher's Note

All claims expressed in this article are solely those of the authors and do not necessarily represent those of their affiliated organizations, or those of the publisher, the editors and the reviewers. Any product that may be evaluated in this article, or claim that may be made by its manufacturer, is not guaranteed or endorsed by the publisher.
