# Supplementary material for: “You Never Exhale Fully Because You're Not Sure What's NEXT”: Parents' Experiences of Stress Caring for Children With Chronic Conditions
Source: Front Pediatr. 2022 Jun 27;10:902655. doi: 10.3389/fped.2022.902655 (PMC9271768; doi:10.3389/fped.2022.902655)
Supplement: Supplementary file 1 [file Data_Sheet_1.PDF]

Supplementary Material

1 Supplementary Table: Interview guide

**Opening Question**	1. Please tell me about [child's name] in your own words.<br>• Prompts: How does the child's long-term condition affect you? Please tell me about how your child/children was/were diagnosed with [long-term condition], or if not yet, please tell me about the diagnosis journey so far? Please tell me how you felt when you were told that your child had [long-term condition(s)]? Any changes from diagnosis to now?
**Main questions**	**Stress**<br>2. Please describe what parenting stress means to you.<br>• Prompts: What causes parenting stress for you? How do you express your stress? Can you please describe a situation when you experienced stress related to the child's condition? How do you think your stress affects the rest of the family/ the child's condition? Please describe your sleep quality.
	**Coping**<br>3. When you experience parenting stress, how do you handle it?<br>• Prompts: Ways of coping /particular strategies for helping you? Self-care? Respite?<br>4. What kind of support would you need to reduce parenting stress?<br>• Prompts: At home versus hospital? What could have been/can be done to reduce parenting stress for you?
	**Relationships**<br>5. How would you describe your relationship with your child?<br>• Prompts: How do you think that [long-term condition] has made a difference to [child's name] and your relationship? Setting influences such as home versus hospital?<br>6. How do you think that [long-term condition] has made a difference to your other relationships?<br>• Prompts: e.g., friendships/colleagues/family members
	**Identity**<br>7. What effect does your child's/children's [long-term condition] have on you?<br>• Prompts: Please describe any differences to how you see yourself?<br>8. What about the way other people see you?
	**Improvements/recommendations to parental stress**<br>9. Please describe any recommendations you feel are needed to aid your experience of stress as [child's name] parent?

**Closing**	10. Is there anything else that hasn't come up that you would like to add?
**Probing questions**	Can you tell me more about…?<br>Can you give an example of…?<br>Can you elaborate on that…?
